# Liberation of recalcitrant cell wall sugars from oak barrels into bourbon whiskey during aging

**DOI:** 10.1038/s41598-018-34204-1

**Published:** 2018-10-26

**Authors:** Jarrad Gollihue, Mitchell Richmond, Harlen Wheatley, Victoria G. Pook, Meera Nair, Isabelle A. Kagan, Seth DeBolt

**Affiliations:** 1 Department of Horticulture, University of Kentucky, Lexington, KY 40546 USA; 2 Department of Plant and Soil Sciences, University of Kentucky, Lexington, KY 40546 USA; 3 Sazurac Buffalo Trace Distillery, 113 Great Buffalo Trace, Frankfort, KY 40601 USA; 4 Kentucky Spirits Research Institute, University of Kentucky, Lexington, KY 40546 USA; 5 USDA-ARS Forage-Animal Production Research Unit, University of Kentucky, Lexington, KY USA; 6 Present Address: Canadian Tobacco Research Foundation, 500 Highway #3, Tillsonburg, ON N4G 4H5 USA

## Abstract

Oak barrels have been used by humans for thousands of years to store and transport valuable materials. Early settlers of the United States in Kentucky began charring the interior of new white oak barrels prior to aging distillate to create the distinctively flavored spirit we know as bourbon whiskey. Despite the unique flavor and cultural significance of “America’s Spirit”, little is known about the wood-distillate interaction that shapes bourbon whiskey. Here, we employed an inverse method to measure the loss of specific wood polysaccharides in the oak cask during aging for up to ten years. We found that the structural cell wall wood biopolymer, cellulose, was partially decrystallized by the charring process. This pyrolytic fracturing and subsequent exposure to the distillate was accompanied by a steady loss of sugars from the cellulose and hemicellulose fractions of the oak cask. Distinct layers of structural degradation and product release from within the barrel stave are formed over time as the distillate expands into and contracts from the barrel staves. This complex, wood-sugar release process is likely associated with the time-dependent generation of the unique palate of bourbon whiskey.

## Introduction

“It is suggested to me that if the barrels should be burnt upon the inside, say only a 16^th^ of an inch, that it will much improve [the bourbon whiskey]”- a grocer writes to John Corlis, Lexington, KY July 15^th^ 1826^1^.

The cultural practice of aging spirits and wine in charred oak barrels dates back to Roman times and while today’s food and beverage industry has adopted modern technology to increase productivity and improve consistency, the traditional oak barrel is still held in high regard. First selected for their unique physical and chemical properties, barrels constructed from the heartwood of 80- to 120-year-old oak trees (*Quercus*) reliably hold a variety of liquids of variable viscosity^2,3^. In addition, they contribute to the unique flavor that has become characteristic of the wine and spirits stored within, resulting in their continued use^4–8^.

The contribution made by the oak barrel to the flavor of the bourbon whiskey is affected by the cultural practices associated with its production. First of all, the staves of an oak barrel are seasoned in open air. During this time, hydrolyzable tannins are lost and macromolecules, such as lignin, are degraded leading to an increase in aromatic compounds in the wood^9–11^. The staves are then bent into shape assisted by steam. After the barrel is constructed, its interior is charred. Charring, a form of fast pyrolysis, is the process during which the interior of the barrel is exposed to a natural gas flame that reaches 1950 °C, leading to the modification of the biopolymers, cellulose, hemicellulose and lignin, which are the major constituents of oak heartwood^8,11,12^. Each biopolymer can withstand a different intensity of heat, with hemicellulose (30% of the cell wall and dominated by xylan and glucomannan^13,14^) breaking down first at around 225–325 °C, followed by the Β-1,4 linked glucan structure of paracrystalline cellulose at 315–440 °C and finally by lignin, which requires temperatures exceeding 400 °C to induce degradation^15,16^. The barrel is now ready to hold distillate, which will become bourbon only after it has been aged for a sufficient amount of time – a minimum of 24 months for Kentucky straight bourbon whiskey.

The aging of bourbon whiskey takes place in a ‘rickhouse’ - a large multi-tiered warehouse, traditionally made of limestone but presently constructed from modern building materials. Though these structures shield the bourbon barrels from the weather, they are relatively uninsulated and allow for seasonal temperature shifts. The effect that these changes in temperature have on whiskey has been documented with studies demonstrating variation in the amount of sugars, the phenolic content and the accumulation of various other volatile and nonvolatile compounds^17–19^. These changes are thought to be due to the expansion and contraction of the distillate as it heats and cools. When warm, the distillate expands and penetrates the staves of the barrel and when it subsequently cools, components of the wood are drawn from the staves, giving rise to the flavor profile associated with bourbon whiskey.

It is clear that both charring and aging affect the interaction between the oak barrel staves and the distillate^17^. However, the majority of studies to date have focused on the changing flavor profile of the bourbon whiskey. Herein, we investigate the oak cask, focusing on whether recalcitrant cell wall polysaccharides such as cellulose and hemicellulose are extracted during barrel aging. Such extraction would provide an unusual source of chemical building blocks.

## Results

### A nomenclature for the layers within a barrel stave

**Figure 1:**
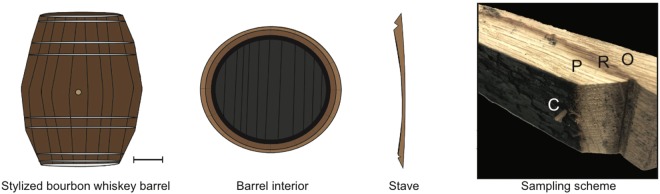
The composition of a bourbon whiskey barrel. Diagrams showing a stylized bourbon whiskey barrel (scale bar: 15 cm), a cross section of the charred barrel interior and a stave. The sampling scheme highlights the charred surface of the barrel stave (C); the inner portion of wood, which undergoes thermal degradation and distillate penetration (P); the red line (R), which indicates the depth to which the distillate penetrated the stave; and the outer portion of the stave (O). Note that the thickness of the red line and its distance from the outside of the stave vary both within the stave and among staves.

Standard bourbon whiskey barrels are built to hold 53 gallons or 200 liters of distillate and are composed of a series of narrow staves held together by metal rings (Fig. 1)^17,18^. The process of charring a barrel followed by the aging of a spirit held within it results in barrel staves with four distinct layers, for which no satisfactory nomenclature currently exists. To address this, we established a simple nomenclature for these layers in an effort to maintain a repeatable sampling protocol for barrel staves (Fig. 1).

The innermost layer of the barrel stave is exposed directly to the flame during charring and therefore undergoes pyrolysis (Supplementary Fig. 1A). We refer to this section as the ‘C layer’. It contains active carbon from combustion, which is documented to help remove sulfur and impurities from spirits^8^. The depth of the C layer is dependent upon the charring duration. Though not charred, the next layer of the stave is subjected to thermal degradation^20^. Herein denoted as the ‘P layer’, this wood has a visibly lighter appearance after whiskey has matured in the barrel (Fig. 1 comparing P versus O) due to the extraction that has occurred during the aging process^21^. The most striking visual change in the barrel stave is the appearance of the red line that occurs after whiskey maturation. The red line is the spirit penetration mark, which likely indicates to what depth the distillate has penetrated, marking the end of the P layer. This section of the stave is here referred to as the ‘R layer’. The position and breadth of the red line varies considerably across staves and even within a single stave, highlighting the limitations of sampling schemes that rely only on depth (Supplementary Fig. 1D)^5^. Finally, the ‘O layer’ is the outer section of the stave. This section of wood has not undergone thermal degradation or extraction via spirit penetration.

The thermal degradation and extraction that occurs during charring and aging in the C and P layers of the whiskey barrel are hypothesized to influence the release rate of ‘wood sugar’ entering the ethanol during aging. Cellulose and hemicellulose, both composed of monosaccharides, are postulated to be the source of wood sugars, but how these biopolymers are broken down and to what extent their breakdown products enter the whiskey within the barrel is unknown.

### Charring and aging reduced cellulose crystallinity

Cellulose is a dominant metabolic component of all tree stem tissue, and this remains true for the white oak (*Quercus alba*) barrel stave. Comprising highly ordered repeating β-1,4-glucan units, cellulose has a crystalline structure that is resilient to chemical or enzymatic deconstruction^15,16,21^. However, bourbon barrels are required to be charred as defined by the standard of identity, and this cast modification is initiated by a natural gas flame (1950 °C) applied directly to the inner surface of the barrel – a temperature well in excess of that which is needed to break down cellulose. The duration of this process varies with producer but is generally between 15 and 45 seconds, resulting in a range of charring grades^12^. The charring grade to which the barrels used in this study were subjected was #4, which is approximately 45 sec. This charring is similar to pyrolysis without atmospheric modification and could therefore alter the cellulose present in the barrel staves.

**Figure 2:**
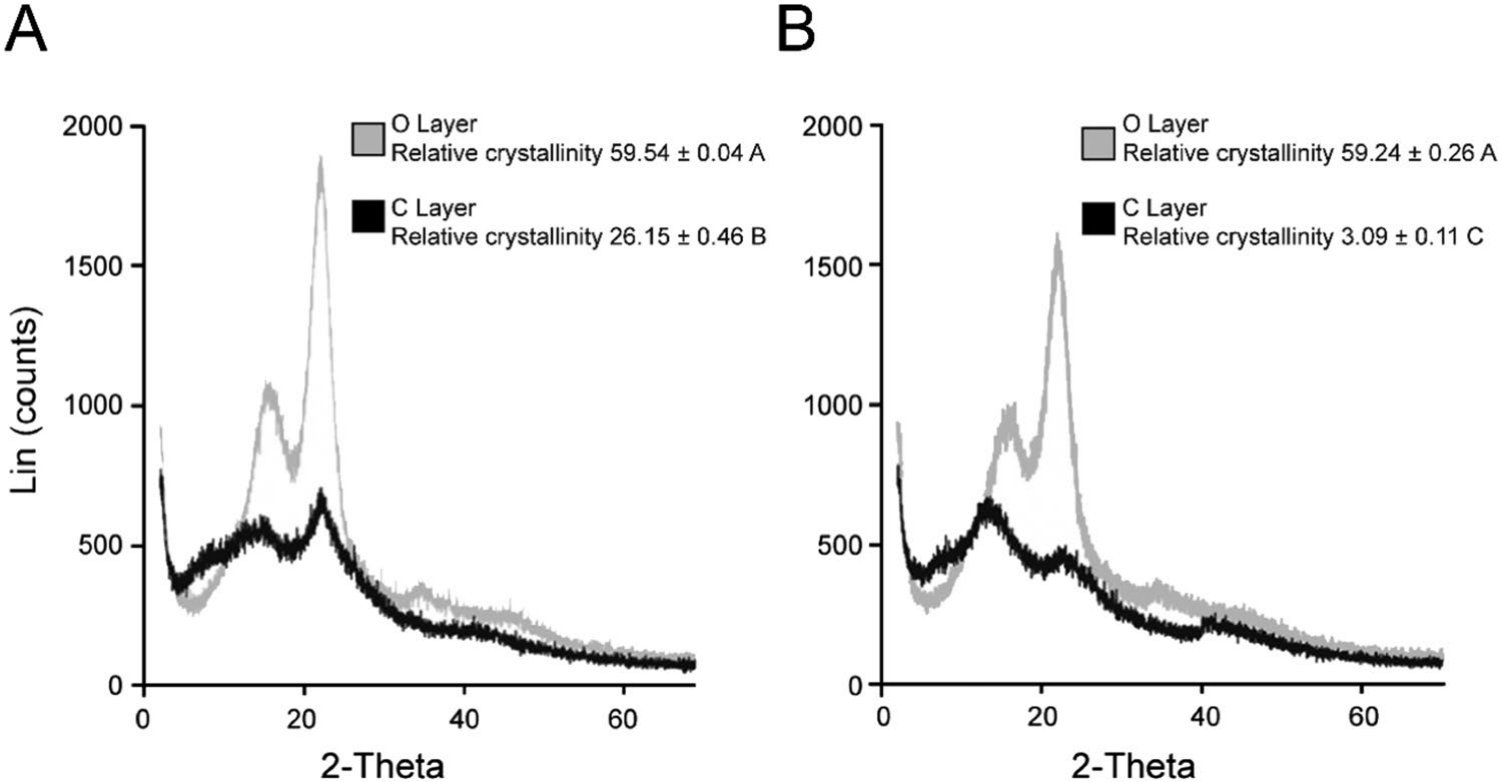
Cellulose crystallinity is reduced as a result of charring. The crystallinity of cellulose in the C layer and the O layer was measured by X-ray scattering in a new barrel (**A**) and a ten-year-old barrel (**B**). The C layer of both barrels shows a significant reduction in relative crystallinity. The relative crystallinity of the O layer was similar in both the new barrel and the ten-year-old barrel, whereas the crystallinity of the C layer was significantly lower in the ten-year-old barrel (Tukey-Kramer multiple comparison test, P < 0.05, n = 3; different letters denote significant differences).

The intermolecular forces acting within cellulose in its native state lead to crystallization^15^. This crystallized structure makes cellulose difficult to break down, as only the outermost portions of the cellulose strand provide accessible reaction sites. However, we found that the process of charring reduced cellulose crystallinity. Using wide-angle X-ray diffraction and Bragg-Brentano geometries (symmetrical reflection), we demonstrated that the relative crystallinity index (RCI) of cellulose in the C layer of a new barrel stave was decreased by 50% compared with wood from the O Layer (Fig. 2A). This loss of crystallinity was similar to that found in samples of wood subjected to 300 °C for one hour^22^. A comparative analysis of a ten-year-old barrel indicated that the crystallinity of cellulose in the O layer remained constant over time. This constancy was in contrast with the C layer, which displayed an even greater reduction in cellulose RCI after ten years of whiskey maturation, dropping to 3.1° 2θ from 26.1° 2θ in a newly charred stave (Fig. 2B). The P and R layers of the ten-year-old barrel both had mean RCI values of approximately 60. These values were not statistically different (P = 0.962, two-way ANOVA) from those of the O layers of a new barrel, indicating that the crystallinity of the cellulose in the interior of the barrel stave was not affected by whiskey maturation (Supplementary Information Table 1).

### Alterations in cellulose visualized through confocal microscopy

**Figure 3:**
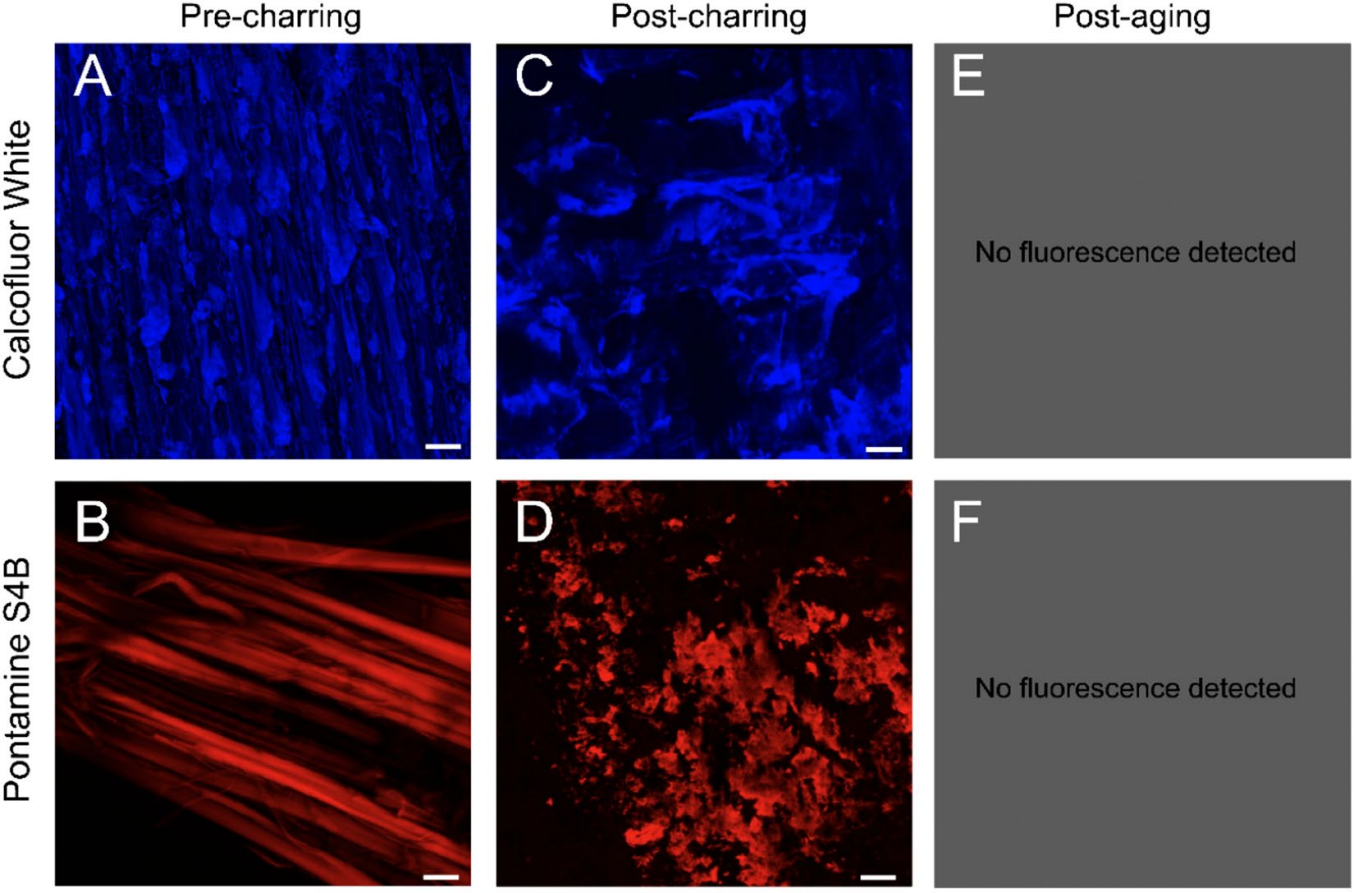
β-1,4-glucans and crystalline cellulose are disrupted by charring and are eliminated after aging from the C Layer. Confocal microscopy images of the C layer stained with Calcofluor White to show β-1,4-glucans (**A**,**C**,**E**) and Pontamine S4B to show crystalline cellulose (**B**,**D**,**F**). Panels A and B show the presence of β-1,4-glucans and crystalline cellulose in the C layer before the stave has been charred. Panels C and D show that disruption of β-1,4-glucans and crystalline cellulose occurred during charring. In panels E and F, no fluorescence was detected, indicating a loss of β-1,4-glucans and crystalline cellulose from the C layer following aging.

In addition to measuring the relative crystallinity of cellulose in the wood tissue, we found that cellulose in barrel staves could be visualized through confocal microscopy of wood samples stained with the fluorescent cellulose binding agents Pontamine S4B, which is specific for crystalline cellulose, and Calcofluor White, a broader cellulose reporter. Optical evaluation of stave samples was used to reveal whether any changes in cellulose content were evident. In an un-charred barrel stave, the cellulose matrix appeared as a series of linear striations of cellulosic material (Fig. 3A,B). These were consistent in form across both histochemical tests but were clearer with the crystalline cellulose interactive dye Pontamine S4B. In contrast, the process of charring influenced the form of the cellulose polymorphs. Inspection via confocal microscopy revealed a distinct morphological change in the organization of cellulose after charring has taken place (Fig. 3C,D). This change was best described as a mottling of the linear structures observed in untreated oak (Fig. 3A). Though we observed changes in the appearance of the cellulose polymorphs, before and after charring, the histochemical assessment supported the presence of intact dye-reactive cellulose. In this same charred layer, we saw strong interactions with the distillate. After whiskey maturation had taken place for a period of five, six or ten years (data presented for the ten-year-old barrel staves only, but consistent results were observed for all maturation periods), no detectable fluorescence arising from cellulose in the C layer was observed (Fig. 3D,E). This loss of cellulose is explored further in the following section.

### Whiskey maturation led to a reduction in the cellulose content of the barrel stave

Little is known about the chemistry of whiskey during maturation. The transfer of oak components derived from the barrel into aged distillate is known to impart 50–80% of the flavor but is only beginning to be explored^21^. In terms of chemical building blocks derived from the wood, cell wall polysaccharides are the major available substrates present. Based on the reduced cellulose crystallinity in the charred oak fraction of the barrel, we questioned whether exposure to distillate, which is added to the barrel at exactly 62.5% ethanol at Buffalo Trace Distillery (Frankfort, KY, USA), would cause a slow but measurable solvent based deconstruction of cellulose from the charred stave.

**Figure 4:**
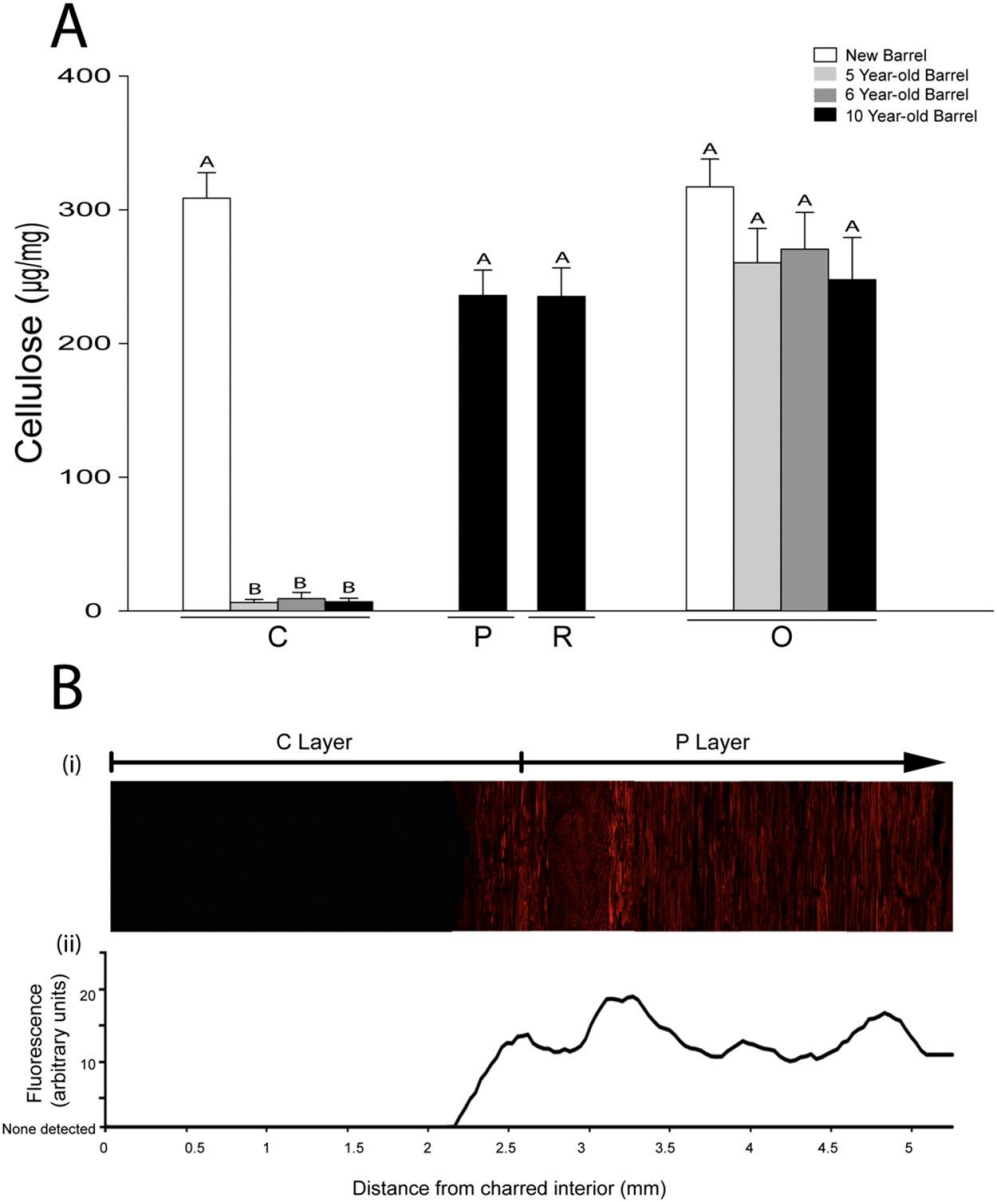
Cellulose is lost from the C layer after aging. (**A**) The cellulose content of the C and O layers of a new barrel, a five-year-old barrel and a six-year-old barrel, and the C, P, R and O layers of a ten-year-old barrel were measured using values that were obtained by the Updegraff method and are presented as micrograms of cellulose per milligram of barrel material. A Tukey-Kramer multiple comparison test was used to determine statistical significance (n = 12; different letters denote statistically significant differences at P < 0.05; error bars indicate standard error). (**B**) The cellulose content gradually increases with distance from the charred interior of the ten-year-old barrel stave. Confocal microscopy image using Pontamine S4B to stain crystalline cellulose (**i**). The lack of crystalline cellulose in the C layer can be seen by the absence of fluorescence. Relative fluorescence was measured across the image showing a gradual increase in crystalline cellulose with distance from the charred interior (**ii**).

We found a significant reduction in the cellulose content of the charred portion of the barrel staves, with 6–9 µg/mg present in the C layer of five-, six- and ten-year-old barrel staves compared to the 235–317 µg/mg of cellulose found in the C layers of new staves, the P and R layers of the ten-year-old barrel staves and O layers of all barrel staves (Fig. 4A; P < 0.0001, two-way ANOVA). We also found a degree of variation between barrels that was not statistically significant.

Given that the thermal degradation occurring in the C layer of the barrel stave is not uniform, but rather more intense in the interior of the barrel, we hypothesized that the cellulose content of the C layer after whiskey maturation would also exhibit variation which was not revealed through our layer-by-layer analysis. We therefore used confocal microscopy to image a barrel stave stained with Pontamine S4B, generating optical cross sections of a barrel stave to reveal the presence or absence of crystalline cellulose. A gradual increase in crystalline cellulose occurred through the C layer which is tracked in Fig. 4B(i). We detected no fluorescence in the first 2.2 mm of the C layer, (Fig. 4B(ii)**)**, beyond the background pixel noise in the micrographs. Using the same technique but staining with Calcofluor White enables detection of β-1,4-glucans, the components of cellulose. We found that after charring, the presence of β-1,4-glucans of the C layer was comparable to the other layers of the stave (Supplementary Fig. S2A) whereas after aging there was a visible drop in fluorescence detected in the C layer, indicating that these β-1,4-glucans may be extracted from the wood (Supplementary Fig. S2B).

### Hemicellulose polysaccharides declined as a result of charring

In contrast to the homogeneity of cellulose, hemicellulose constitutes a group of biopolymers. In hardwoods, hemicellulose comprises around 30% of the cell wall and is dominated by xylan and glucomannan^13,14^. The chemical composition of hemicellulose results in a polymer that is amorphous and more likely than cellulose to be chemically and thermally degraded at lower temperatures (~200 °C)^23^. The term ‘wood sugar’, when used to describe the flavor profile of whiskey, is generally thought to derive from the hemicellulose present in the barrel staves.

**Figure 5:**
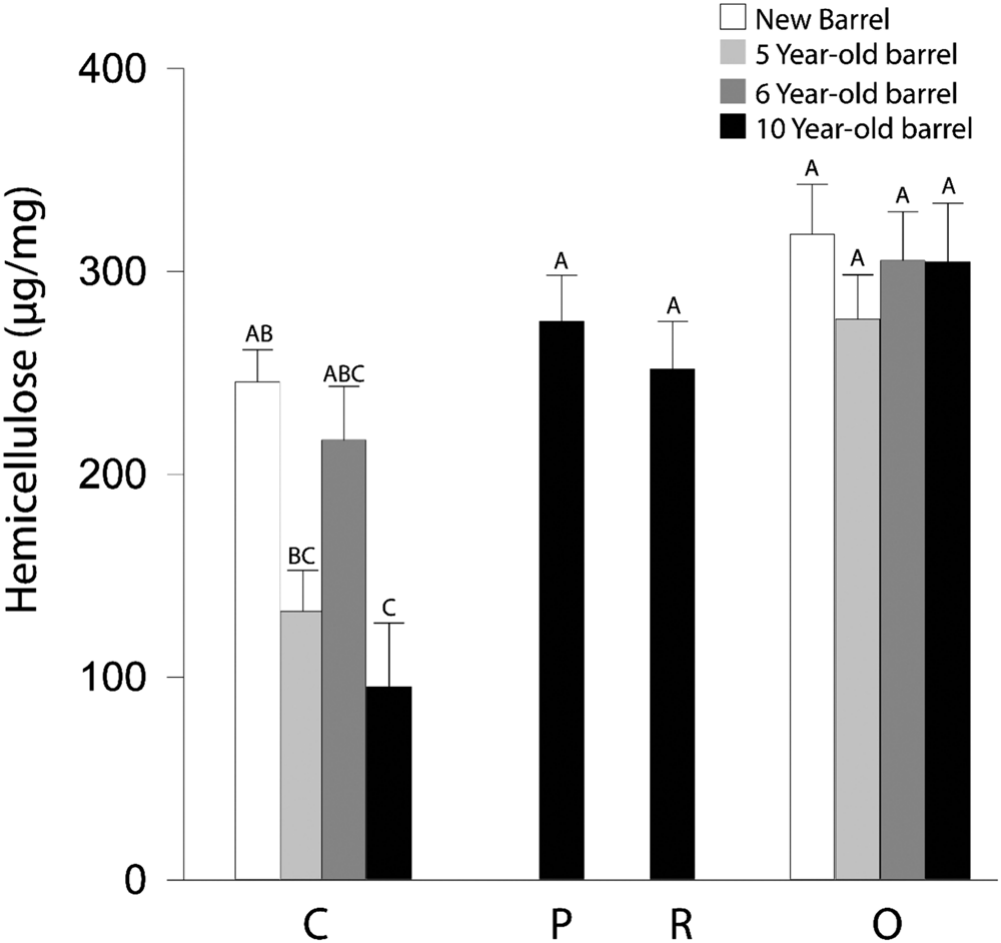
Hemicellulose content declines during aging in the C layer. For the C and O layers of a new, five-, six-, and ten-year-old barrel, and for the P and R Layers of a ten-year-old barrel, neutral monosaccharides were measured by HPLC-PED after hemicellulose hydrolysis with TFA. Bars represent the sum of all measured monosaccharides. Charring induced a slight reduction in hemicelluloses which was not statistically significant. Whiskey maturation appears to have a greater effect on the hemicellulose content with the C layer of the ten-year-old barrel exhibiting a significant reduction compared with a new barrel stave. A Tukey-Kramer multiple comparison test was used to determine statistical significance (n = 12; different letters denote statistically significant differences at P < 0.05; error bars indicate standard error).

**Figure 6:**
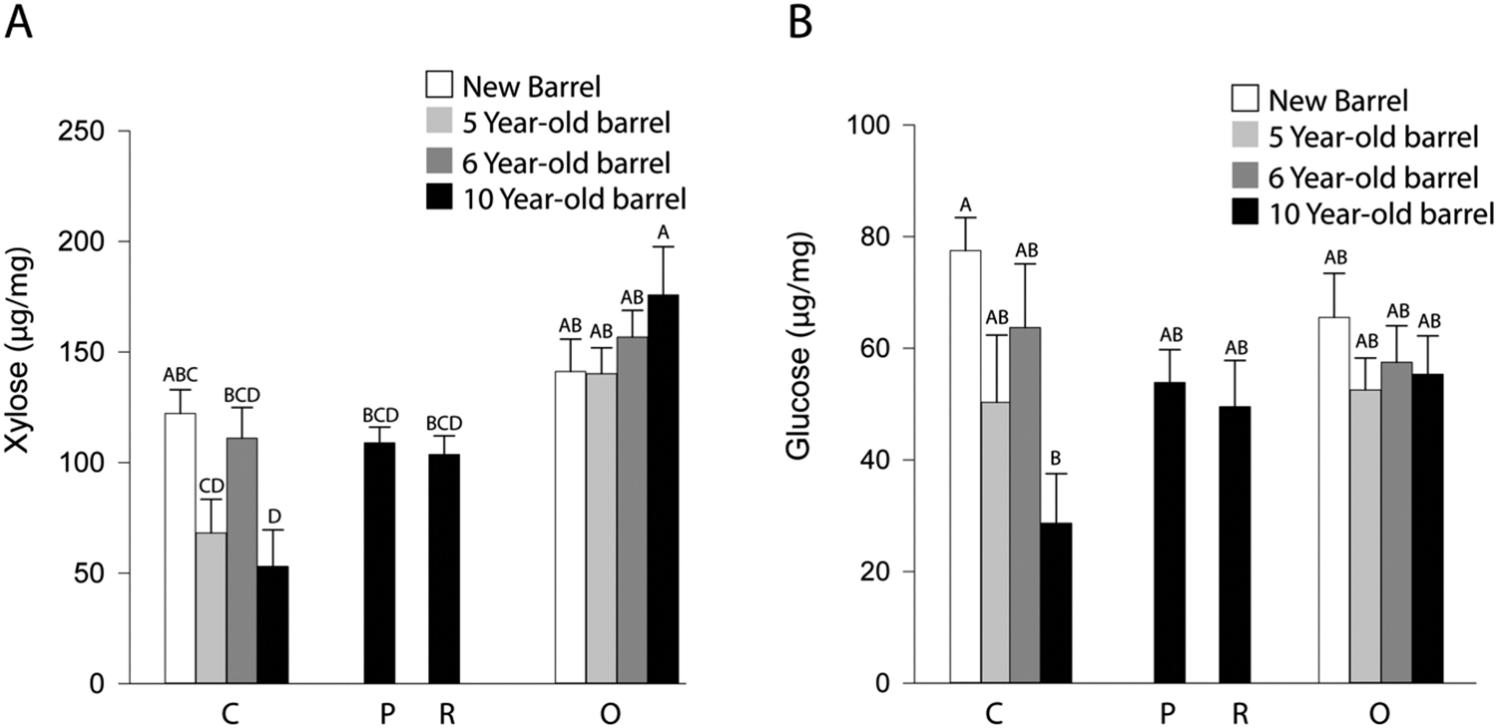
The effect of charring and aging on the xylose and glucose content of barrel staves. The xylose and glucose content of the C and O layers of new, five- and six-year-old barrel staves and the C, P, R and O Layers of staves from a ten-year-old barrel were measured using HPLC-PED after hydrolysis with TFA. (**A**) Xylose content was unaltered by charring and showed a significant decrease after ten years of aging with distillate. (**B**) Glucose levels were unaltered by charring as well, although the C layer of the new barrel was numerically higher in glucose than the O layer. Aging led to a reduction in glucose in the C Layer of the ten-year-old barrel. Different letters indicate statistically significant differences (Tukey-Kramer multiple comparison test, P < 0.05, n = 12).

We carried out a similar suite of analyses for hemicellulose as for cellulose and found that the charring process reduced neutral sugars as a whole but this reduction was not significant, specifically when looking at O and C layer of a new barrel (Fig. 5). This result was surprising given the susceptibility of hemicellulose to thermal degradation. We therefore examined the quantified individual monosaccharides comprising the total neutral sugars (Fig. 6 and Supplementary Table S2) and found that the level of glucose in the C layer of new barrel staves was higher than that of the O layer numerically but did not have a statistical separation (Fig. 6). We hypothesize that the glucose found in hemicellulose is being thermally degraded but is then being replaced with glucose from the decrystallized cellulose in the C layer.

Hemicellulose content was found to decrease as a result of aging, though the reduction was only significant in the C layer of the ten-year-old barrel (Figs 5 and 6**)**. The high level of hemicellulose seen in the six-year-old barrel was numerically but not statistically different from the hemicellulose level in the C layers of the other aged barrels (Fig. 5). The numerical variation may indicate an underlying complexity to hemicellulose hydrolysis during whiskey maturation. We believe it is no coincidence that one of the major carbohydrates found in bourbon whiskey, xylose, is also the monosaccharide that is present in the largest amount in the hemicellulose of oak heartwood^17,24,25^. We therefore quantified both xylose and glucose, the other major carbohydrate found in bourbon whiskey. Figure 6A shows that, like hemicellulose as the same pattern appears, charring reduced the xylose content by a numerical amount with mean amounts of xylose being 141 µg/mg and 122 µg/mg in O and C Layers, respectively, of new barrel staves (P < 0.0001, two-way ANOVA).

We also saw a significant decline in xylose in the C layer of ten-year-old barrel staves (Fig. 6a). Glucose was significantly reduced in the C layer of the ten-year-old barrel staves but not in the C layer of the five- and six-year-old barrel staves (Fig. 6B). The levels observed in the P, R and O layers were not statistically different after charring and aging (Fig. 6). These results indicate that the combined charring and aging processes play a role in the incorporation of wood sugars into bourbon whiskey. The remaining hemicellulose sugars measured can be found in Supplementary Table 2.

In order to further technically validate these data, a sulfuric acid hydrolysis method for sugar analyses was performed (see methods), which provided similar results.

The composition results found above suggested that cellulose and hemicellulose in American oak barrel staves used for bourbon whiskey production have a different composition than the native wood^26^. The results also indicated that cellulose was being degraded in the C layer of the oak cask during whiskey maturation. Hence, we cross referenced our results using a different method by surveying five additional new barrel staves and five additional ten-year-old barrel staves. The additional method used 72% sulfuric acid hydrolysis of barrel material for total glucose content and 4% sulfuric acid hydrolysis for hemicellulose sugar content. Sugars were then separated by high performance liquid chromatography (HPLC) and detected by pulsed electrochemical detection (PED). The findings for cellulose mirrored those obtained by the Updegraff method, in that charring did not change cellulose content and that whiskey maturation degraded the content of cellulose found in the C layer of the barrel (Supplementary Fig. 3). Cellulose values in the O layers of the new and ten-year-old barrel were found to be 288 to 298 µg/mg, similarly to the previous analysis (Supplementary Table 4 and Fig. 3). In contrast to the previous result, obtained using the TFA method, neutral sugar levels were found to be lower after charring, with significantly less hemicellulose in the C than in the O layer of a new stave (Supplementary Fig. 4). Aging led to a further numerical reduction in hemicellulose in the ten-year-old barrel when compared to the new barrel but this effect was non-significant (Supplementary Fig. 4). These data support a difference in the results of the two methodologies, as found in prior studies^27–29^. Supplementary Fig. 5A shows that, as with hemicellulose as a whole, charring resulted in a significant decline in the level of xylose with means of 244 µg/mg and 78 µg/mg in O and C layers of new barrel staves, respectively (P < 0.0001, two-way ANOVA). Again, xylose declined in the C layer of aged barrel staves but not significantly. In contrast, a significant increase in glucose was induced by charring (means of 56 and 27 µg/mg in the C and O layers of new barrel staves, respectively) followed by a significant reduction after aging in the ten-year-old barrel staves which brought the glucose content to 30 µg/mg (Supplementary Fig. 5B). The levels observed in the P, R and O layers were not statistically different after charring and aging (Supplementary Fig. 4). The measured sugar values can be found in Supplementary Table 3.

### A cellulose degradation model system

The results detailed above indicate that cellulose is being broken down during charring and aging and that its constituent sugars are being transferred to the bourbon whiskey. An impractical but potentially insightful experiment that could test this hypothesis would involve observing the fate of radiolabeled cellulose within oak trees made into barrels. Given the limitations of such an approach we sought to develop a model system that could be used to test our hypothesis in the laboratory.

We chose wood chips that were milled to sawdust as the model substrate and used 62.5% ethanol as our model spirit. The sawdust was first toasted at 200 °C before the aging process began. This low toasting temperature was chosen firstly as a way of emulating a barrel environment without thermally degrading the cellulose in the sawdust (cellulose is thermally degraded at temperatures between 315 °C and 440 °C^30^). Charring was not employed as a cultural treatment. Toasting temperatures used in the cooperage industry are highly variable and depend on both the producer’s preference and the intended use of the barrel. They range from 47 °C to 235 °C^7^, but 200 °C for one hour was deemed a plausible temperature based on a series of experimental studies (data not presented). The material was treated in this manner to ensure that any loss of cellulose from the wood could be attributed to a cellulose-ethanol interaction. In addition, toasting was used in place of charring to ensure reproducibility.

**Figure 7:**
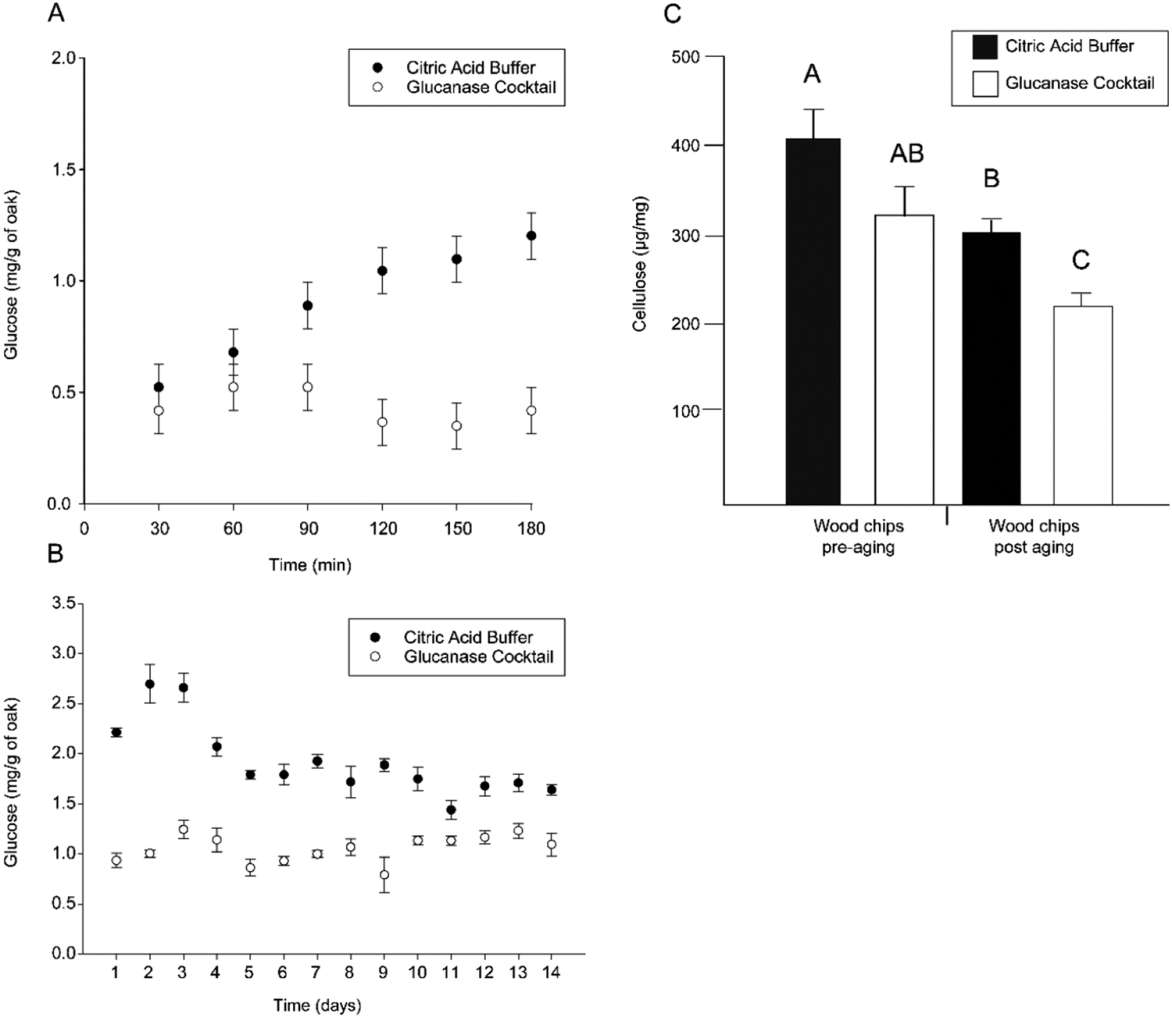
A model experiment demonstrates that the source of the glucose found in the distillate is cellulose. (**A**) Levels of glucose in a model distillate were measured every 30 min for 180 min during aging with a model substrate (wood chips) that had been treated with either a control solution (citric acid buffer; black dots) or a glucanase cocktail (white dots) (error bars are SEM). After 60 min, the level of glucose in the distillate interacting with the wood chips with reduced cellulose content was significantly lower than the control and remained so throughout the experiment. While not shown the treatment and control were statistically different with a 95% confidence interval at all time points. (**B**) The above experiment was carried out for a further 14 days, during which time the glucose levels of each of the model spirits were measured daily. In the system with the low-cellulose model substrate, glucose was significantly lower than in the control system throughout the experiment. While not shown the treatment and control were statistically different with a 95% confidence interval at all time points except at day 11. (**C**) Levels of cellulose in the model substrate that had been treated with a control solution (citric acid buffer; black bars) or a glucanase cocktail (white bars) before and after aging with a model distillate. The level of cellulose in the substrate treated with the glucanase cocktail was significantly lower than that of the control, and the levels of cellulose in both treatments showed a significant reduction after aging with the model spirit (Tukey-Kramer multiple comparison test P < 0.05, n = 6).

After toasting, the sawdust and the ethanol were then placed in sealed glass vessels and maintained at 55 °C in order to quicken the aging process to a time period conducive to study. Our aim for this study was two-fold. Firstly, we wished to examine any correlation between the cellulose content in a model substrate and the glucose content of an aged model spirit. Secondly, we hoped to investigate whether the amount of cellulose present in the substrate affected the rate or the total amount of glucose released over time. In order to achieve our second goal, we modified the cellulose content of the model substrate using a cocktail of endo- and exoglucanases prior to toasting and aging. We then measured the glucose content of the model spirit solution every 30 minutes for the first 180 minutes of the aging process and then once per day for 14 days (Fig. 7A,B).

At the start of the experiment, both sawdust treatments produced model spirits with similar amounts of glucose (Fig. 7A). However, after 60 min the model spirits began to diverge, with the difference in the level of glucose increasing for the entire 180 min period. Differences peaked at two days (Fig. 7B), when the model spirit aged with buffer-treated sawdust had more than 2.5 mg/g of glucose, while the model spirit aged with saccharified sawdust had less than 1.5 mg/g of glucose. The glucose content of the model spirit aged with buffer-treated sawdust then began to decline until Day 11, where it appeared to level off at 1.5 mg/g. This decline may be due to the breakdown of glucose via catalysis, which results in a variety of compounds not measured here^31,32^. The glucose content of the model spirit produced by the saccharified sawdust remained relatively constant after the initial increase to approximately 1.3 mg/g. After the experiment, the cellulose content of the sawdust was measured again, and both treatments revealed a similar magnitude of reduction in cellulose (Fig. 7C).

## Discussion

The cultural practices employed in the bourbon whiskey industry expose barrel staves to environmentally variable conditions. Months of weathering followed by periods of steaming, toasting and charring, culminating in years of whiskey maturation, produces barrel staves that bear little resemblance to their parent tree. Herein, we examined the chemical composition of bourbon whiskey barrel staves at different stages on the production line. We found that both the cellulose and the hemicellulose of the barrel stave were affected by charring and aging in a quantitative manner and as a function of their interaction with distillate. We also developed a model spirit maturation system in the laboratory to validate that cellulose is extracted during the aging process and contributes to the glucose-based building blocks within the aged distillate. We conclude that cell wall sugars are a major source of building blocks in bourbon whiskey.

The synthesis of cellulose, a biopolymer bound tightly by inter- and intra-molecular hydrogen bonds and van der Waals forces, results in anisotropic cellular expansion, leading to the upright growth of a tree. Due to its paracrystalline structure, cellulose is recalcitrant to chemical and enzymatic degradation, a property which has led to the assumption that this biopolymer is not broken down and released during whiskey production^32^. Our data did not support this assumption. Rather, we demonstrated that the crystallinity of cellulose in the barrel staves was reduced by the charring process. A comparison of the C layer and the O layer of a new barrel stave indicated that pyrolytic disruption is responsible for a reduction in RCI (Fig. 2).

In Figs 2–4 we showed that aging further reduced cellulose crystallinity in addition to reducing the cellulose content in the C layer. The weakly acidic environment created by the whiskey may be sufficient to cause the secondary reduction in cellulose crystallinity over extended periods of time. We hypothesize that once broken down, the cellulose may be transferred from the barrel stave into the distillate as a result of seasonal temperature fluctuations which cause the bourbon whiskey to expand into and contract from the barrel staves, drawing with it the constituents of cellulose from the C layer^33^. The loss of cellulose from the C layer after time-dependent exposure to distillate contrasted with the presence of cellulose in the C layer in a new barrel. One possible explanation of the preservation of the cellulose in the C layer of a new barrel stave is that charring partially decrystallizes the cellulose microfibrils, but does not denature them. The heating rate of the stave during charring and steaming may also be slightly buffered by the dimensions of the stave. Within barrel staves, the effects of pyrolysis decrease with larger material, which generally causes fragmentation of the cell wall, rather than other pyrolysis reactions from the deconstruction of the material^34^. Variables may also influence cellulose breakdown, such as maturation conditions, the position of the barrel within the rickhouse, natural variation among barrel staves or subtle differences in the original distillate.

Interestingly, we found the O layer of barrel staves comprises 30% cellulose, whereas the reported cellulose content of white oak is 42%^26^, and we found that oak staves from a stave maker (Independent Stave, Lebanon, KY) that had not undergone steaming and charring were also 42% cellulose. Though we cannot be certain of how the cellulose in the O layers of the barrel staves is being degraded, it is feasible that it is due to the effect of barrel processing and whiskey maturation. Firstly, barrel staves are weathered for up to 36 months (24 months at Buffalo Trace Distillery) and then bent into shape using steam at a temperature and duration that varies with producer. Steaming is known to alter the cell wall biopolymers in wood and the susceptibility of cellulose to enzymatic/acidic saccharification^35^. Therefore, steaming alone could be sufficient to cause a reduction in the recalcitrance of cellulose, and the combined effects of weathering and steaming could alter the composition of the oak in the barrel staves^35–37^. There are also biotic and abiotic factors that occur during aging and that could contribute to this alteration^38^. Finally, the cellulose in the O layer may be available to the whiskey molds that grow on the exterior of the barrels and in and around rickhouses^39^. Future work is need to explore these hypotheses.

The pattern of degradation of hemicellulose was found to be different to that of cellulose. In Fig. 5 we show that overall, hemicellulose was degraded during the maturation process, and a more complex pattern of deterioration emerged from whiskey maturation, rather than a simple loss over time that was expected from previous work on carbohydrate accumulation in bourbon whiskey^17,24^. Hemicellulose decreased during aging, to an extent that was significant in the ten-year-old barrel C layer when compared to the new C layer. As during whiskey maturation the volume in the barrel decreases, it is plausible that hemicellulose hydrolysis is not uniform across the barrel^8^. However, when examining glucose alone, we saw a numerical increase in levels of the C layer of a new barrel that was not statistically significant. This could be explained by pyrolysis resulting in the reduction of cellulose crystallinity, yielding a more amorphous glucose polymer that could be degraded by a dilute acid^40^. Prior research has shown that under thermal analyses, degradation dynamics of hemicellulose and cellulose differ, with the weight loss of hemicellulose occurring at 220–315 °C compared with 315–400 °C for cellulose^16^. Thus, after charring we may see the formation of a number of hemicellulose breakdown products such as furans and acetic acid^41^.

The pattern of degradation of hemicellulose was found to be complex as values differed across the two neutral sugar quantification methods, TFA and sulfuric acid, but similar trends emerged. As outlined above, many factors could influence the carbohydrate composition of barrel wood in which hemicellulose would be altered. Therefore, the variation observed in the levels of hemicellulose could partially be explained by stave-to-stave variation from the barrel production process or alternatively, by a difference in hydrolysis conditions resulting from the different acids used^27^. Sulfuric acid hydrolysis can be problematic as the process can destroy the extracted sugars. TFA, however, does not have this risk, but rather, incomplete hydrolysis can occur^28,29^. The difference between the acids used in the two methods may explain why the levels of rhamnose, arabinose, galactose, glucose and mannose were higher in the data generated by TFA hydrolysis, while xylose levels were higher after sulfuric acid hydrolysis (Supplementary online Dataset 1).

The difference in the degradation dynamics of the two biopolymers may be due to their structure. Cellulose has a secondary crystalline structure which increases its thermal stability compared to the relatively amorphous hemicellulose^16^. Therefore, under particular thermal conditions, hemicellulose will be degraded into a variety of products, while cellulose will lose its crystallinity but the polymer will remain intact^16,42,43^. However, the temperature range under which these changes would occur is limited. Though it is possible that barrel charring occurs within this temperature range, we do not yet fully understand how the heat is transferred through barrel staves during this process.

Our data suggest that wood derived cell wall sugars are quickly liberated from the more complex polysaccharide pools in the C layer of the oak. This sugar release occurred in two categories: (1) cellulose was decrystallized by charring and degraded after interaction with distillate, and (2) hemicellulose was degraded by whiskey maturation, though the degradation was significant only in 10-year-old barrels. Using a model barrel system we demonstrated that it is possible that these wood sugars enter the whiskey and then are likely broken down catalytically into a variety of compounds that have been previously studied^27^. By extrapolating the sugar units present in the C layer, defined as the innermost 2.2 mm (Fig. 4B(ii)**)**, we found that the quantity of glucose building blocks that could be released from an oak cask into the whiskey held within is equivalent to 300 g from the C layer alone. This amount would result in 1.3 g/L of glucose for a standard sized barrel - a greater amount than estimated in previous indirect studies^17,24^. This difference may be explained by the acidification of whiskey that occurs during maturation^17^. Acidification of an ethanol solution produces a reaction environment that allows the esterification of cellulose. The esterification of cellulose with ethanol can produce an ethyl-glucoside product that first degrades to become 5-hydroxymethylfurfural and then further degrades to myriad other compounds in an ethanol environment^31,32,44,45^. However, this reaction has not been shown to occur in a whiskey solution.

The data presented here indicate that hemicellulose and cellulose are released from oak barrels during cultural treatment and whiskey aging respectively. Our results may provide an explanation as to why congeners change when barrels are re-used for scotch whisky or other end uses, as the carbohydrate pools appear to be depleted during bourbon whiskey aging^17^. Hemicellulose content in the C layer of the barrel yielded results that are difficult to interpret, with levels of individual monosaccharides varying with the method used. This variability may indicate that hemicellulose hydrolysis is more complex in bourbon whiskey maturation than simple extraction over time or that the variation within barrel material exceeds the limit of our sampling and analytical methods. These results were produced from barrels that were aged in Frankfort, KY, USA over ten years and are not the product of a controlled laboratory experiment. Thus, these data reveal where variation exists between barrel staves, consistent with cooperage production practices that use staves from different silvicultural zones.

Barrels by their production process exhibit considerable variation and have been a topic of much discussion in the literature and in the alcoholic beverage production industry. Barrel variation is a product of the combined effects of natural variation in individual oak trees, environmental variation affecting forests, variation in weather during the stave seasoning period, non-uniform charring and toasting procedures, and variation in temperature within the rickhouse during maturation^10,11,46–49^. Our ability to understand how these factors affect the final product is impeded by the lack of information regarding the chemical processes that occur during whiskey maturation, and it is the goal of this study to provide some fundamental data on processes at play in the oak barrel during whiskey aging^17^.

## Materials and Methods

### Chemicals

All chemicals and reagents used were of analytical grade or higher. Chemicals were obtained from Sigma Aldrich (St Louis, MO) as applicable.

### Wood samples

In order to investigate the effect of whiskey maturation on barrel staves, samples of wood were taken from new staves that had been constructed into a barrel, then disassembled (provided by the Independent Stave Company, LLC) or staves that had been part of a barrel used to age whiskey (provided by Buffalo Trace Distillery, Frankfort, KY). All staves were composed of American white oak (*Quercus alba*) and processed using the same methods prior to barrel construction. A total of five staves were sampled per treatment with the ten-year-old staves originating from two different barrels and five new staves. Sampling also included another barrel that aged bourbon for six years along with a five-year-old barrel that had been used to age rye whiskey; these barrels were sampled by scraping off the C and O layers of five barrel staves instead of breaking them down. For proprietary reasons it is not possible to disclose the exact mash bill for the distillate stored in the barrels.

Both charring and aging modify the wood of the barrel stave. However, the degree to which the wood is modified depends firstly on the distance from the charred interior of the barrel and secondly on the depth to which the distilled spirit penetrates the stave. Since charring is a highly regulated procedure, the depth of the C layer is fairly uniform across all staves. However, the penetration of the distilled spirit varies greatly among staves and even within a stave. Though highly variable, this depth is easily tracked by the red line that is produced in the stave (Fig. 1), here referred to as the R layer. Therefore, unlike previous work, we did not sample the staves based on depth. Rather, a 20 cm long section of each layer (see Fig. 1) was shaved off using a wood gouge (Pfeil #7, 35 mm) according to the methods described by Doussot *et al*.^10^. The samples were then dried for 24 hours at 100 °C and homogenized using a grinder equipped with a 1 mm sieve (Arthur H. Thomas Co. Scientific, Phila, PA).

### Quantification of hemicellulose content via TFA

The hemicellulose fraction of each sample was determined by 2 N trifluoroacetic acid hydrolysis at 121 °C for 1 hr followed by HPLC separation and detection by PED according to Foster *et al*., Rocklin and Pohl, and Rocklin *et al*.^50–52^. Approximately 5 mg of barrel alcohol insoluble material was subjected to TFA hydrolysis conditions previously described, with the addition of glucosamine as an internal standard. The resulting monosaccharides from the hydrolysis were then quantified with a correction of response factors from monosaccharide standards of different concentrations to allow area to be converted to molar amounts. The monosaccharides measured were normalized to the amount of barrel material used in the sample preparation.

### Cellulose and Neutral Sugars analysis via Sulfuric Acid Hydrolysis

Barrel samples were digested by sulfuric acid hydrolysis as described by Yeats *et al*.^53^. Neutral sugars were separated by high-performance liquid chromatography (HPLC) coupled to pulsed electrochemical detection (PED)^51,52^. Samples (25 µL) were separated on a CarboPac PA1 column (Thermo Fisher Scientific, Waltham, MA, USA). The CarboPac PA1 guard column and analytical column were 50 and 250 mm long, respectively, and both had an internal diameter of 4 mm. The HPLC employed was an ICS-5000 + model (Thermo Fisher Scientific). The HPLC method was based on that of Downie and Bewley^54^, with the following modifications: the flow rate was 0.8 mL/min; the column temperature was 26 °C; sugars were eluted isocratically with 22 mM sodium hydroxide (NaOH) for 36 min; and the column was subsequently washed for 6 min with 200 mM NaOH in 1 M sodium acetate, flushed 12 min with 200 mM NaOH, and reequilibrated for 13 min with 22 mM NaOH. Sugars were identified by comparing peak retention times with those of the commercial standards: fucose, rhamnose, arabinose, galactose, glucose, mannose, and xylose (Sigma-Aldrich, St. Louis, MO, USA). Peaks were integrated with Chromeleon 6.8 software (Thermo Fisher Scientific). Sugars were quantified with calibration curves of 0.05 to 5 µg/mL of each standard. For glucose, dilutions ranged from 0.05 to 10 µg/mL.

### Confocal microscopy

Dried wood samples stained with Calcofluor and Pontamine S4B were mounted in culture dishes with a coverslip bottom (MatTek). Once mounted, stave samples were imaged in darkness, after being exposed to Calcofluor White for 5 min^55^. Imaging was performed on an Olympus FV1000 laser scanning confocal microscope using a 60X N.A 1.4 water-immersion objective. The microscope is equipped with lasers for excitation wavelengths ranging from 405–633 nm; Calcofluor White was visualized at 405 nm. All image processing was performed using Olympus Fluoview software (Olympus) and ImageJ (W. Rasband, National Institutes of Health, Bethesda, MD). Relative fluorescence was calculated by measuring pixel density in ImageJ.

### Quantification of cellulose crystallinity by X-ray scattering

Samples consisted of biomass that had been oven-dried at 60 °C for 36 hours. Tissue was then homogenized using a grinder equipped with a 1 mm sieve (Arthur H. Thomas Co. Scientific, Phila, PA). Biomass samples were then contained in a custom-built Biomass Crystallinity sample holder of pressed boric acid as described in Harris *et al*.^56^. A Bruker-AXS Discover D8 Diffractometer (Bruker-AXS USA, Madison, WI) was used for wide angle X-ray diffraction with Cu Ka radiation generated at 30 mA and 40 kV. The experiments were carried out using Bragg-Brentano geometries and diffractogram data were collected between 2° and 70° or 2° and 40° (for samples with little baseline drift), with 0.02° resolution and 2 s exposure time interval for each step (run time, 2 h). The data analysis was carried out using the calculation for relative crystallinity index. Data were examined in Diffrac-Plus-XRD Commander (BrukerAXS, Karlsruhe, Germany), EVA and TexEval (Bruker-AXS, Karlsruhe, Germany) software.

### Model maturation System

*Quercus alba* heartwood was purchased from a commercial supplier. This wood was milled to a particle size of 250 μm and washed with a citrate buffer (0.05 M, pH 4.8) or a citrate buffer and an enzymatic cellulase solution at 0.3%. The sawdust solution was incubated at 50 °C for 72 hours to remove cellulose under agitation. After digestion, the glucose content of the solution was measured. Digested wood chips were strained using a wood mesh and washed with deionized water until no glucose could be detected. Washed wood chips were dried in an oven for 24 h at 50 °C then toasted at 200 °C for 1 h. After toasting, 10 g of wood chips were placed a jar with 200 mL of 62.5% ethanol. Jars were placed in an incubator at 55 °C and shaken at 100 rpm. Glucose content was measured at 30-minute intervals over 360 minutes. This series of measurements was repeated every 24 hours over 14 days. Glucose content was determined using a glucose analyzer that had been calibrated with a glucose standard curve.

### Colorimetric analysis of cellulose content

Samples were incubated at 70 °C with 70% ethanol for one hour a total of three times resulting in the production of alcohol insoluble residue (AIR). Five milligram samples of AIR were then boiled in acetic-nitric acid reagent (with a ratio of acetic acid:nitric acid:water of 8:1:2) for 30 min. The resulting material was washed three times with 8 mL water and 4 mL of acetone and dried under a vacuum for 48 hours. Samples were then hydrolyzed in 67% sulfuric acid for one hour^57^.

The glucose content was determined using the anthrone method^57^. Briefly, 20 µL of sulfuric acid hydrolyzed sample was mixed with 500 μL water to 1 mLof 0.3% anthrone with concentrated sulfuric acid on ice. Absorbance was then measured at 620 nm using a spectrophotometer (Bio-Mate Thermo Fisher Scientific, Waltham, MA). The cellulose content was calculated by multiplying the measured glucose concentration of each sample by the total volume of the assay and then by a hydration correction factor of 0.9 to correct for the water molecules added during hydrolysis of the cellulose polymer.

### Statistical analysis

Data were subjected to a two-way ANOVA test using JMP 11. Means were separated using a Tukey’s Multiple Comparison Test at alpha = 0.05. The software JMP®, Version11. SAS Institute Inc., Cary, NC, 1989–2007 was used for all statistical calculations.

## Electronic supplementary material

Supplementary online information

Supplementary online dataset 1
