# Supplementary material for: Liberation of recalcitrant cell wall sugars from oak barrels into bourbon whiskey during aging
Source: Sci Rep. 2018 Oct 26;8:15899. doi: 10.1038/s41598-018-34204-1 (PMC6203734; doi:10.1038/s41598-018-34204-1)
Supplement: Supplementary file 1 — Supplementary online information [file 41598_2018_34204_MOESM1_ESM.docx]

**Online Supplementary Information**

Liberation of recalcitrant cell wall sugars from oak barrels into bourbon whiskey during aging

Jarrad Gollihue^1^, Mitchell Richmond ^2, μ^, Harlen Wheatley^Ψ^, Victoria G. Pook^1,4^, Meera Nair^1^, Isabelle Kagan^5^, and Seth DeBolt^1,4^

^1^ Department of Horticulture, University of Kentucky, Lexington, KY, 40546

^2^ Department of Plant and Soil Sciences, University of Kentucky, Lexington, KY, 40546

^3^ Sazurac Buffalo Trace Distillery, 113 Great Buffalo Trace, Frankfort, KY, 40601

^4^ Kentucky Spirits Research Institute, University of Kentucky, Lexington, KY, 40546

^5^ USDA-ARS Forage-Animal Production Research Unit, University of Kentucky, Lexington, KY

Correspondence should be addressed to [sdebo2@uky.edu](mailto:sdebo2@uky.edu)

^μ^ Current Address: Canadian Tobacco Research Foundation, 500 Highway #3, Tillsonburg, ON, N4G 4H

Contents:

Supplementary Tables S1-5

Supplementary Figures S1-5

**Supplementary Table S1. Relative crystallinity index (RCI) of the P, R and O layers of a ten-year-old barrel**. The RCI of the P, R and O Layers of the ten-year-old barrel were not different from each other or from the O layer of the new barrel. The mean is calculated from three replicates. Std Error = standard error.

|  | Relative crystallinity index mean (%) | Std Error |
| --- | --- | --- |
| New Barrel |  |  |
| O layer | 59.54 | 0.04 |
| 10-year-old Barrel |  |  |
| P layer | 60.19 | 0.02 |
| R layer | 59.55 | 0.27 |
| O layer | 59.24 | 0.26 |

**Supplementary Table S2. Barrel oak neutral sugar composition as determined by digestion with trifluoroacetic acid.** Observed mean values of rhamnose, arabinose, glucose, galactose, xylose, mannose and total sugars reported as μg per mg of barrel material. Monosaccharide values were obtained from digestion with 2 M trifluoroacetic acetic acid and separation by HPLC-PED. Values represent the average of 12 replicates. Std Err = standard error.

|  | Rhamnose (μg mg-1) | | Arabinose  (μg mg-1) | | Galactose  (μg mg-1) | | Glucose  (μg mg-1) | | Xylose (μg mg-1) | | Mannose  (μg mg-1) | | Total sugar  (μg mg-1) | |
| --- | --- | --- | --- | --- | --- | --- | --- | --- | --- | --- | --- | --- | --- | --- |
| Layer | Mean | Std Err | Mean | Std Err | Mean | Std Err | Mean | Std Err | Mean | Std Err | Mean | Std Err | Mean | Std Err |
| 0C | 4.4 | 1.2 | 2.8 | 1.4 | 19.7 | 5.4 | 77.5 | 5.9 | 122.1 | 10.7 | 18.2 | 4.06 | 245.6 | 15.7 |
| 5C | 0.9 | 0.1 | 0.5 | 0.2 | 6.0 | 0.7 | 50.3 | 12.0 | 68.1 | 15.1 | 6.1 | 1.29 | 132.4 | 20.2 |
| 6C | 1.7 | 0.4 | 1.6 | 0.8 | 21.2 | 8.2 | 63.7 | 11.4 | 110.9 | 13.9 | 16.9 | 0.86 | 216.8 | 26.5 |
| 10C | 0.6 | 0.3 | 1.5 | 1.0 | 7.5 | 6.0 | 28.6 | 8.8 | 52.9 | 16.5 | 3.3 | 2.81 | 95.1 | 31.5 |
| 10p | 9.3 | 0.9 | 6.9 | 0.5 | 88.6 | 13.0 | 53.8 | 5.9 | 108.8 | 7.1 | 7.1 | 2.19 | 275.5 | 22.5 |
| 10R | 8.2 | 0.7 | 8.3 | 0.5 | 74.2 | 9.8 | 49.5 | 8.3 | 103.5 | 8.5 | 7.3 | 2.00 | 252.0 | 23.3 |
| 0O | 9.0 | 1.4 | 7.3 | 1.5 | 80.1 | 11.1 | 65.5 | 7.8 | 141.2 | 14.6 | 13.6 | 2.07 | 318.3 | 24.6 |
| 5O | 7.3 | 1.0 | 7.8 | 1.1 | 53.5 | 5.1 | 52.5 | 5.7 | 140.1 | 11.7 | 13.9 | 1.77 | 276.6 | 21.7 |
| 6O | 8.8 | 0.9 | 6.6 | 1.3 | 53.6 | 9.1 | 57.5 | 6.5 | 156.7 | 12.0 | 21.0 | 5.18 | 305.4 | 24.1 |
| 10O | 5.5 | 1.1 | 7.7 | 1.6 | 49.9 | 8.1 | 55.3 | 6.8 | 175.8 | 21.8 | 9.3 | 2.73 | 304.8 | 28.8 |

**Supplementary Table S3. Barrel oak neutral sugar composition as determined after digestion with sulfuric acid.** Observed mean values of rhamnose, arabinose, glucose, galactose, xylose, mannose and total sugars reported as μg per mg of barrel material. Monosaccharide values were obtained after digestion with sulfuric acid and separation by HPLC-PED. Values represent the mean of 10 replicates. Std Err = standard error.

| Year | Layer | **Rhamnose**  **(μg mg-1)** | | **Arabinose**  **(μg mg-1)** | | **Galactose**  **(μg mg-1)** | | **Glucose**  **(μg mg-1)** | | **Mannose**  **(μg mg-1)** | | **Xylose**  **(μg mg-1)** | | **Total sugars**  **(μg mg-1)** | |
| --- | --- | --- | --- | --- | --- | --- | --- | --- | --- | --- | --- | --- | --- | --- | --- |
|  |  | Mean | Std Err | Mean | Std Err | Mean | Std Err | Mean | Std Err | Mean | Std Err | Mean | Std Err | Mean | Std Err |
| 0 | C | 0.1 | 0.1 | 0.9 | 0.5 | 8.8 | 3.8 | 56.1 | 9.8 | 3.2 | 2.2 | 78.6 | 31.5 | 148.0 | 37.4 |
|  | O | 5.6 | 0.8 | 8.3 | 1.4 | 41.1 | 7.9 | 27.9 | 4.7 | 5.3 | 1.2 | 244.5 | 18.1 | 333.0 | 25.4 |
| 10 | C | 0.0 | 0.0 | 0.2 | 0.2 | 0.6 | 0.6 | 30.7 | 7.1 | 0.4 | 0.4 | 20.8 | 12.2 | 52.9 | 19.6 |
|  | P | 8.1 | 0.9 | 8.8 | 1.3 | 55.3 | 9.0 | 26.2 | 4.0 | 10.4 | 3.9 | 256.4 | 25.7 | 365.5 | 28.3 |
|  | R | 7.4 | 1.0 | 12.3 | 0.9 | 40.0 | 3.9 | 26.9 | 5.9 | 10.2 | 4.0 | 291.6 | 31.5 | 388.5 | 40.0 |
|  | O | 7.1 | 0.9 | 8.1 | 0.5 | 37.6 | 3.5 | 26.9 | 3.6 | 9.5 | 3.9 | 300.6 | 25.4 | 390.0 | 30.1 |

**Supplementary Table S4. The cellulose content of barrel staves obtained after digestion via sulfuric acid** The cellulose content of the C and O layers of a new barrel and the C, P, R and O layers of a ten-year-old barrel were measured after two different hydrolysis one with 72% and 4% sulfuric acid. Values are presented as micrograms of cellulose per milligram of barrel material. Std Err = standard error.

| Year | Layer | Cellulose (μg mg-1) | |
| --- | --- | --- | --- |
|  |  | Mean | Std Err |
| 0.00 | C | 272.5 | 20.2 |
|  | O | 288.2 | 24.7 |
| 10.00 | C | 18.4 | 6.6 |
|  | P | 269.3 | 25.6 |
|  | R | 272.9 | 32.4 |
|  | O | 298.0 | 24.5 |

**Supplementary Table S5. The cellulose content of the C and O layers of a new barrel, a five-year-old barrel and a six-year-old barrel, and the C, P, R and O layers of a ten-year-old barrel** These values were obtained using the Updegraff method and are presented as micrograms of cellulose per milligram of barrel material. Std Err = standard error.

| Year | Layer | Cellulose  (μg mg-1) | |
| --- | --- | --- | --- |
|  |  | Mean | Std Err |
| 0 | C | 308.0 | 19.0 |
|  | O | 317.0 | 20.8 |
| 5 | C | 6.2 | 2.3 |
|  | O | 260.4 | 25.5 |
| 6 | C | 9.1 | 4.6 |
|  | O | 270.5 | 27.5 |
| 10 | C | 6.8 | 2.6 |
|  | P | 235.9 | 18.9 |
|  | R | 235.2 | 21.2 |
|  | O | 247.7 | 31.6 |

**
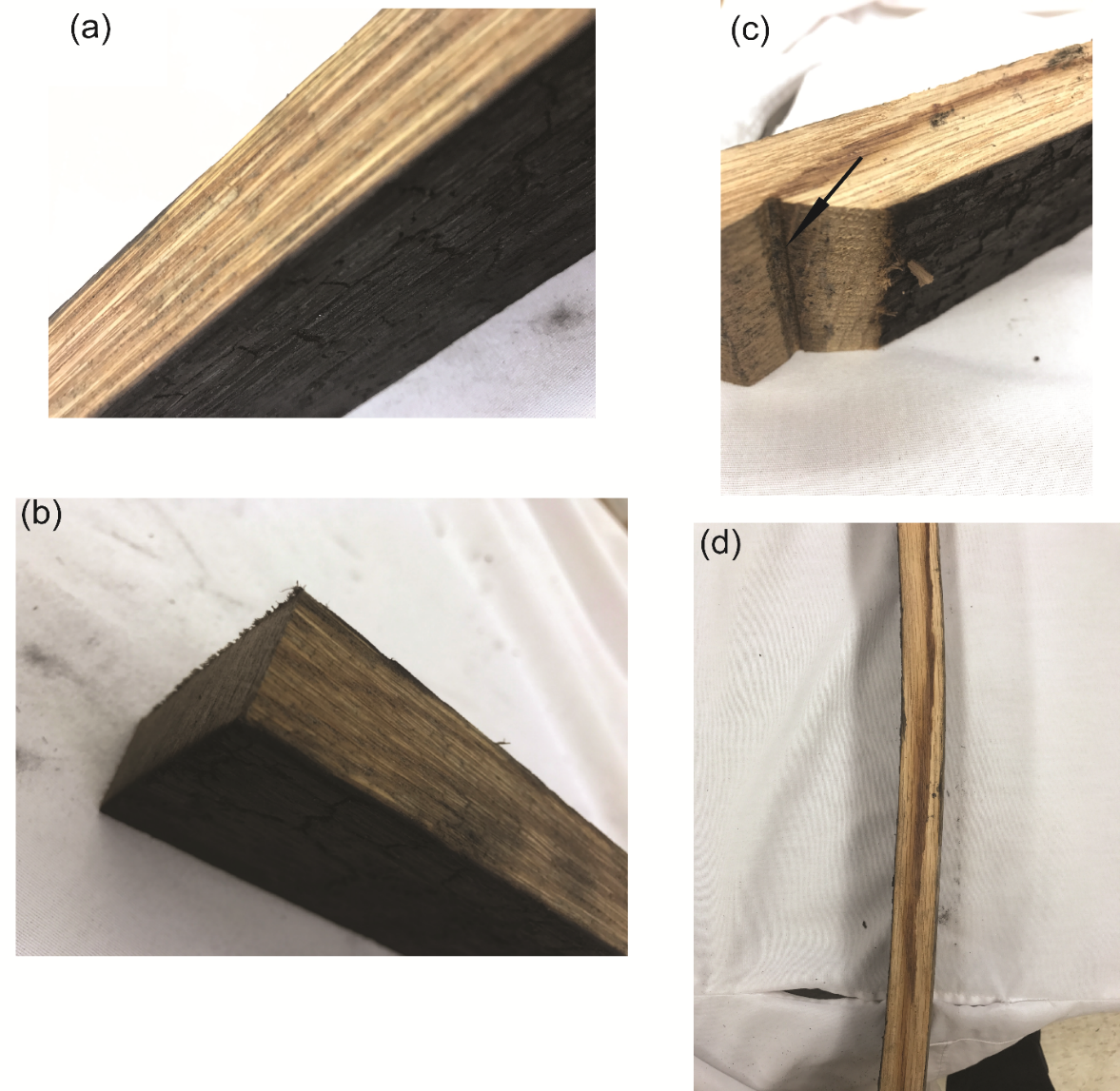
**

**Supplementary Figure S1. The physical appearance of barrel staves is altered as a result of whiskey maturation.** **A-B** A newly charred stave showing the wood grain and the C layer. **C** A barrel stave that has undergone the aging process exhibits the R layer. This image also shows the croze groove (indicated by the arrow) in which the barrelhead is placed in a finished barrel. **D** The depth of the R layer varies considerably in both distance from the outside of the stave and thickness within each barrel stave and among staves.


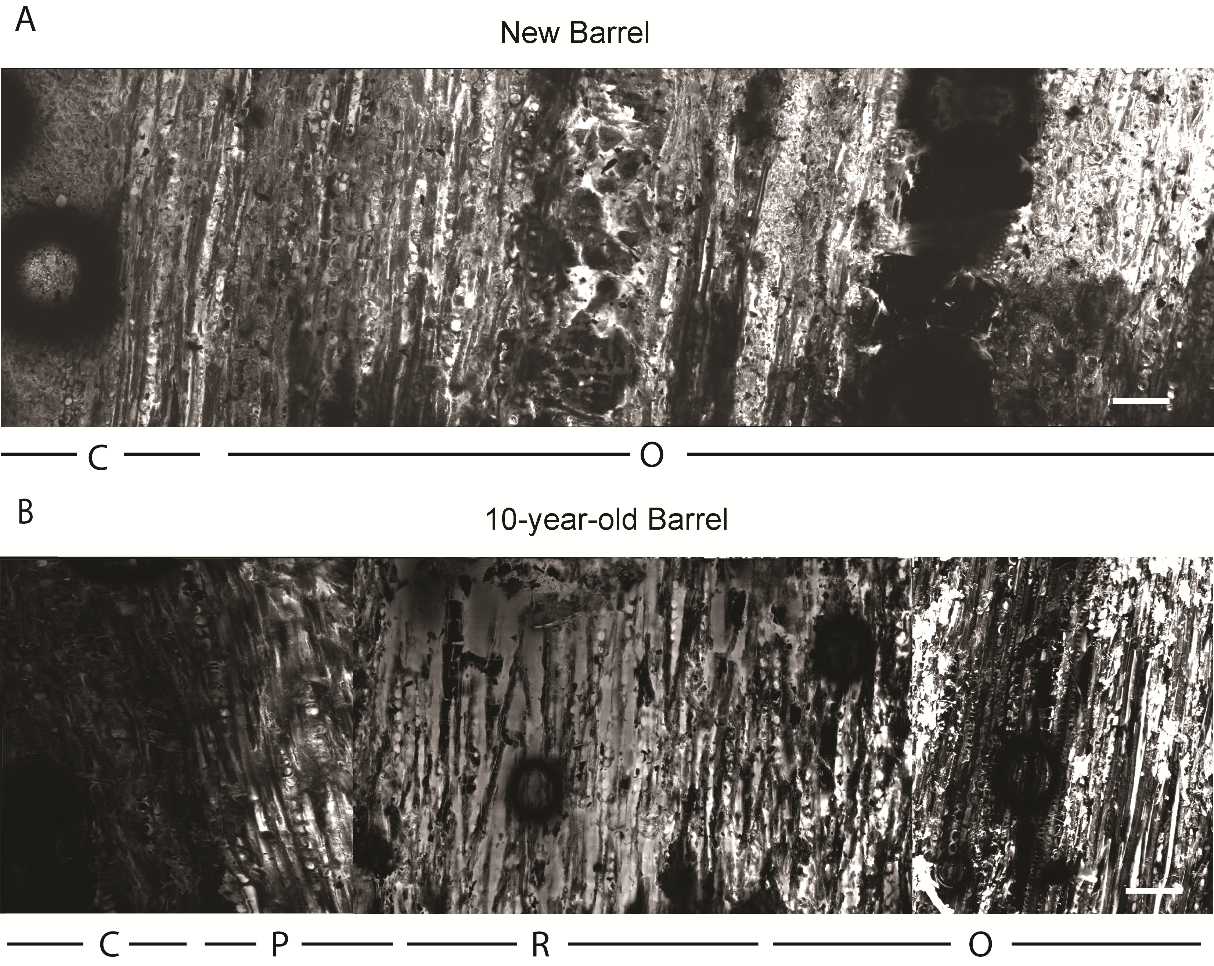


**Supplementary Figure S2. Glucans content across a barrel stave visualized through confocal microscopy.** Cross sections of barrel staves were stained with Calcofluor White. **A** The fluorescence captured from a new barrel stave is fairly consistent through the stave indicating that glucose levels are similar throughout. **B** The C Layer exhibits less fluorescence than the P, R and O layers of the ten-year-old stave indicating that this layer has less glucose. Scale bar = 100μm.

**

**

**Supplementary Figure S3. Cellulose is lost from the C layer after aging. A.** The cellulose content of the C and O layers of a new barrel and the C, P, R and O layers of a ten-year-old barrel were measured using sulfuric acid digestion followed by glucose quantitation via HPLC-PED. Values presented in are μg per mg of barrel material. Measured cellulose content gave a result similar to that determined by the Updegraff method, namely, that the cellulose content of the C layer is unaltered by charring. Different letters indicate statistically significant differences (Tukey-Kramer multiple comparison test, P < 0.05, n=10).




**Supplementary Figure S4. Hemicellulose content declines as a result of charring in the C layer.**

The total neutral monosaccharides content of the C and O layers of a new barrel and the C, P, R and O layers of a ten-year-old barrel using HPLC-PED after hydrolysis with 4% sulfuric acid by taking the sum of all measured monosaccharides (**Fig.** **5**). Hemicellulose values from sulfuric acid hydrolysis indicate that charring significantly reduced the hemicellulose content. There is a further reduction following aging though this is not statistically significant. Hemicellulose content was not reduced by whiskey maturation in the P and R layers of the barrel. Hemicellulose concentrations in the P, R and O layers in the ten-year-old barrel were higher than those found using the TFA method. Different letters indicate statistically significant differences (Tukey-Kramer multiple comparison test, P < 0.05, n=10).


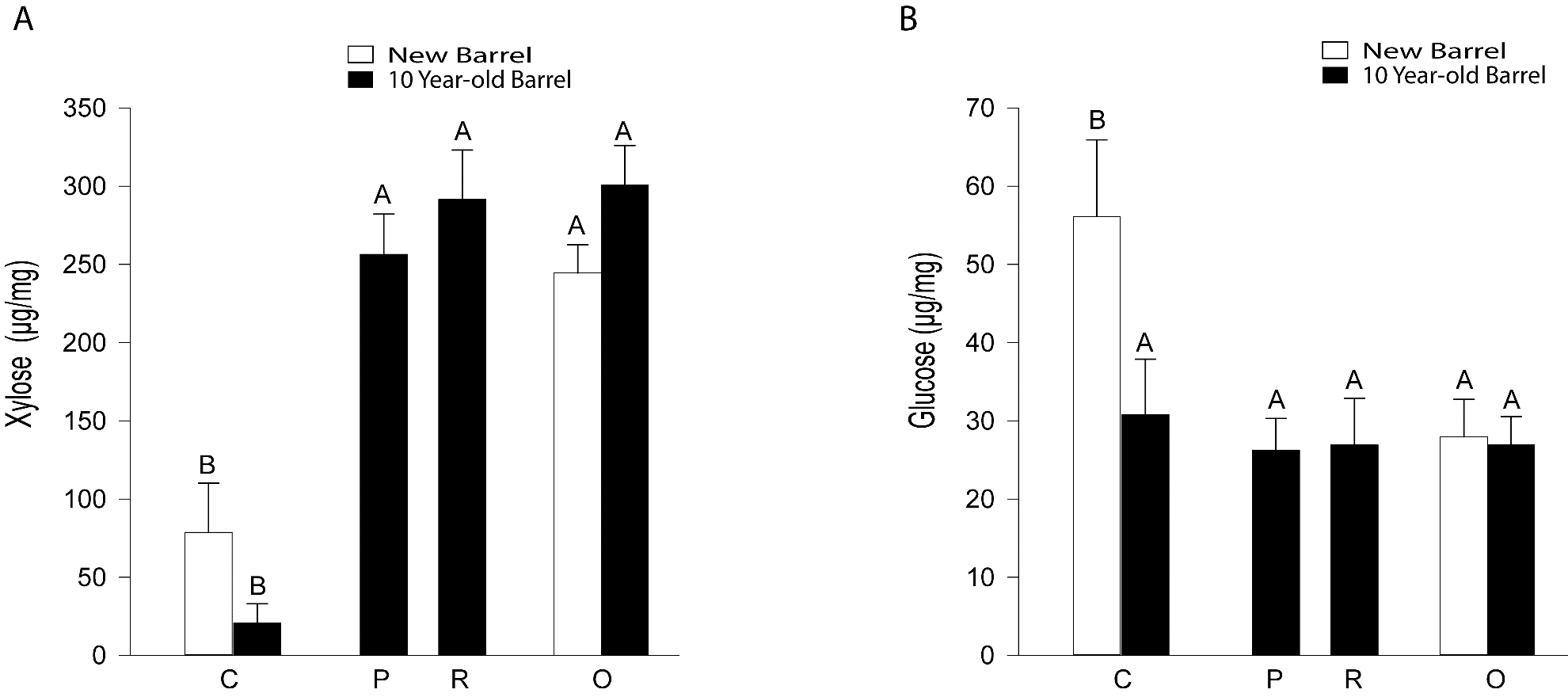


**Supplementary Figure S5 The effect of charring and aging on the xylose and glucose content of barrel staves.** The total neutral monosaccharide content of the C and O layers of new barrel staves and the C, P, R and O layers of staves from a ten-year-old barrel were measured using HPLC-PED after hydrolysis with 4% sulfuric acid. **A.** Xylose content was significantly reduced after charring (P<0.001) and there was a further loss after aging with distillate however this was not a significant reduction. The xylose content measured using this method was greater than that found using the TFA method indicating that incomplete hydrolysis of the xylans in oak hemicellulose may have occurred when using the TFA methods. **B.** Charring induced the opposite effect on glucose levels, with the C layer in the new barrel exhibiting significantly higher levels of glucose than the O layer. The measurements indicate that charring produces a spike in the glucose content in the C layer of new barrel staves which is subsequently degraded by whiskey maturation. Glucose concentrations were similar but generally lower than those found when using the TFA method, indicating that sulfuric acid degraded some of the glucose during hydrolysis. Different letters indicate statistically significant differences (Tukey-Kramer multiple comparison test, P < 0.05, n=10).
